# Performance, blood parameters, ruminal fermentation and microbial community of dairy cows supplemented with *Saccharomyces cerevisiae* fermentation product from dry-off to early lactation

**DOI:** 10.1093/jas/skag056

**Published:** 2026-03-04

**Authors:** Yiming Xu, Jianxin Xiao, Yimin Zhuang, Duo Gao, Wen Jiang, Guobin Hou, Xinjie Zhao, Sumin Li, Tianyu Chen, Shangru Li, Siyuan Zhang, Yanting Huang, Shuai Liu, Ilkyu Yoon, Weina Shi, Mengmeng Li, Wei Wang, Shengli Li, Zhijun Cao

**Affiliations:** State Key Laboratory of Animal Nutrition and Feeding, International Calf and Heifer Organization, College of Animal Science and Technology, China Agricultural University, Beijing, 100193, China; Inner Mongolia Youran Dairy Co., Ltd, Hohhot, 010080, China; State Key Laboratory of Animal Nutrition and Feeding, International Calf and Heifer Organization, College of Animal Science and Technology, China Agricultural University, Beijing, 100193, China; Animal Nutrition Institute, Sichuan Agricultural University, Chengdu, 611130, China; State Key Laboratory of Animal Nutrition and Feeding, International Calf and Heifer Organization, College of Animal Science and Technology, China Agricultural University, Beijing, 100193, China; College of Animal Science and Technology, Yangzhou University, Yangzhou, 225009, China; State Key Laboratory of Animal Nutrition and Feeding, International Calf and Heifer Organization, College of Animal Science and Technology, China Agricultural University, Beijing, 100193, China; State Key Laboratory of Animal Nutrition and Feeding, International Calf and Heifer Organization, College of Animal Science and Technology, China Agricultural University, Beijing, 100193, China; State Key Laboratory of Animal Nutrition and Feeding, International Calf and Heifer Organization, College of Animal Science and Technology, China Agricultural University, Beijing, 100193, China; State Key Laboratory of Animal Nutrition and Feeding, International Calf and Heifer Organization, College of Animal Science and Technology, China Agricultural University, Beijing, 100193, China; State Key Laboratory of Animal Nutrition and Feeding, International Calf and Heifer Organization, College of Animal Science and Technology, China Agricultural University, Beijing, 100193, China; State Key Laboratory of Animal Nutrition and Feeding, International Calf and Heifer Organization, College of Animal Science and Technology, China Agricultural University, Beijing, 100193, China; State Key Laboratory of Animal Nutrition and Feeding, International Calf and Heifer Organization, College of Animal Science and Technology, China Agricultural University, Beijing, 100193, China; State Key Laboratory of Animal Nutrition and Feeding, International Calf and Heifer Organization, College of Animal Science and Technology, China Agricultural University, Beijing, 100193, China; State Key Laboratory of Animal Nutrition and Feeding, International Calf and Heifer Organization, College of Animal Science and Technology, China Agricultural University, Beijing, 100193, China; State Key Laboratory of Animal Nutrition and Feeding, International Calf and Heifer Organization, College of Animal Science and Technology, China Agricultural University, Beijing, 100193, China; Diamond V, Cedar Rapids, IA 52404, United States; Diamond V, Cedar Rapids, IA 52404, United States; State Key Laboratory of Animal Nutrition and Feeding, International Calf and Heifer Organization, College of Animal Science and Technology, China Agricultural University, Beijing, 100193, China; State Key Laboratory of Animal Nutrition and Feeding, International Calf and Heifer Organization, College of Animal Science and Technology, China Agricultural University, Beijing, 100193, China; State Key Laboratory of Animal Nutrition and Feeding, International Calf and Heifer Organization, College of Animal Science and Technology, China Agricultural University, Beijing, 100193, China; State Key Laboratory of Animal Nutrition and Feeding, International Calf and Heifer Organization, College of Animal Science and Technology, China Agricultural University, Beijing, 100193, China

**Keywords:** *Saccharomyces cerevisiae* fermentation product, oxidative stress, inflammation, ruminal microbiome, milk yield

## Abstract

Dairy cows experience oxidative stress, inflammation, and immune dysfunction during the transition from dry-off to early lactation. Postbiotics such as *Saccharomyces cerevisiae* fermentation product (SCFP), consisting of nonliving microorganisms with or without their components, have beneficial effects on the production efficiency and immune function of dairy cows. The objective of this study was to evaluate the effects of SCFP on milk production, milk composition, ruminal fermentation, blood metabolites, oxidative status, inflammatory responses, and the ruminal microbial community in Holstein dairy cows supplemented from the day of dry-off through early lactation. Two hundred cows were blocked on the basis of parity, BCS, milk yield, and the time of dry-off and were randomly allocated to specific treatment groups within each block. The treatments included the control group (CON, *n* = 100) receiving basal diets with no SCFP supplementation and the SCFP group (*n* = 100) receiving basal diets supplemented with 19 g/d of SCFP from d −60 to 60 relative to parturition. Milk yield—monitored for all 100 cows per treatment—was tracked until d 140 postpartum. In parallel, ruminal fluid, feces, milk, and blood samples were collected from a subset of cows (*n* = 20/treatment) during the treatment period for further analyses. Data were analyzed via the MIXED procedure in SAS (SAS Institute Inc.). The results revealed that the average milk yield of dairy cows in the SCFP group was greater than that in the CON group (43.93 vs. 42.08 kg/d, *P *= 0.04, *n* = 100) during the treatment period and remained greater (41.92 vs. 39.98 kg/d, *P *= 0.04, *n* = 100) throughout the 140 d postpartum recording period. Cows fed SCFP had significantly lower serum β-hydroxybutyrate and nonesterified fatty acid concentrations than did those in the CON group. Compared with the CON group, the SCFP group presented greater levels of superoxide dismutase and lower malonaldehyde concentrations. The SCFP group also presented a greater total antioxidant capacity prepartum and higher glutathione peroxidase levels postpartum. Additionally, the SCFP group had lower concentrations of proinflammatory factors, such as IL-1β, serum amyloid A, and haptoglobin, throughout the treatment period, indicating a stronger anti-inflammatory capability. Overall, SCFP supplementation improved the ruminal environment, reduced oxidative stress and the inflammatory status, and ultimately increased milk production.

## Teaser

Supplementation of Saccharomyces cerevisiae fermentation product (SCFP) from dry-off to early lactation enhances dairy cows’ milk production, alleviates oxidative stress and inflammation, optimizes ruminal microbial balance, and exerts a sustained positive effect even after supplementation ceases, offering a valuable nutritional strategy for improving transition cow health and productivity.

## Introduction

The dry period, which bridges the gap between lactations, is essential for mammary involution and redevelopment (Putman et al. 2018). During this period, high-yielding dairy cows face several challenges, including the stress of ceasing milking, udder discomfort, and systemic imbalances (Bachman 2002; Gulay et al. 2003). The transition from pregnancy to a new lactation cycle induces significant stress and immunological changes, leading to increased health issues postpartum (Goff and Horst 1997). As cows start to produce more milk, they experience elevated oxidative stress, particularly during the first 9 wk postpartum (Konvičná et al. 2015). Dairy cows are susceptible to oxidative stress both pre- and post-calving. To mitigate this stress and inflammation, there is interest in providing cows with specialized supplements starting at dry-off and continuing until peak milk production (Abuelo et al. 2013).

*Saccharomyces cerevisiae* fermentation product (SCFP), a type of postbiotic, contains *Saccharomyces cerevisiae* biomass and fermentation metabolites generated through proprietary fermentation processes (Carpinelli et al. 2021). Supplementing transition dairy cow diets with SCFP has been shown to enhance immune system function and antioxidant capacity (Yuan et al. 2015a, 2015b). Additionally, SCFP supplementation during lactation reduces oxidative stress and inflammation responses (Carpinelli et al. 2021; Sivinski et al. 2022), ultimately increasing milk production in dairy cows (Acharya et al. 2017; Dias et al. 2018b). To date, research on SCFP supplementation has been performed primarily during the 3–4 wk period immediately preceding and following calving. Therefore, investigating the effects of extended supplementation with SCFP throughout the entire dry period through early lactation is warranted.

Alterations in the ruminal bacterial population play crucial roles in initiating inflammatory responses in dairy cows (Hu et al. 2022). The addition of SCFP to the diet stabilizes ruminal pH, establishing a conducive environment for ruminal fermentation (Dias et al. 2018a), and improving the ruminal microbial community (Mullins et al. 2013; Zhu et al. 2017; Tun et al. 2020; Carpinelli et al. 2021; Halfen et al. 2021). To our knowledge, the effects of SCFP supplementation from the day of dry-off through early lactation on ruminal microbiota species have not been extensively studied.

The objective of this study was to evaluate the effects of SCFP on the oxidative status, inflammation, ruminal microbial community, and production performance of dairy cows from the day of dry-off through early lactation. We hypothesized that adding dietary SCFP, compared with that in cows in the control group, would increase the antioxidant capacity, strengthen immune responses, mitigate excessive inflammation, influence ruminal characteristics, and ultimately improve overall performance. We also hypothesized that the antioxidant and anti-inflammatory effects of SCFP during the transition period could increase the lactation performance of dairy cows after supplementation is stopped.

## Materials and methods

The experiment and animal procedures were performed according to the Guidelines for Care and Use of Laboratory Animals of China Agricultural University (Beijing, China) and approved by the Animal Ethics Committee of China Agricultural University (Approval No. AW42202702-4-1).

### Animals, treatments, and feeding

**Figure 1:**
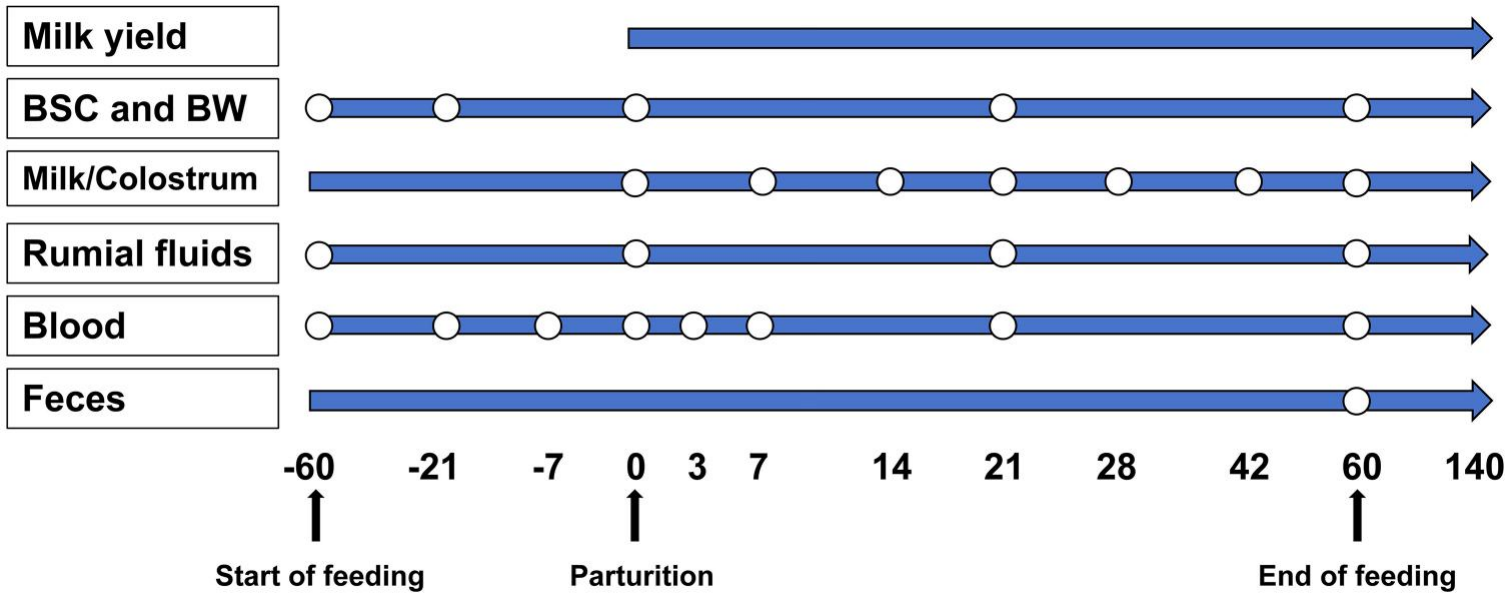
Theoretical timeline of treatment administration and data collection from d −60 to 60 relative to parturition.

This study was conducted at Gansu State Farm Tianmu Dairy Co., Ltd. (Jinchang, Gansu, P. R. China) from June to December 2022. Cows are grouped by physiological stage, with far-off dry cows, close-up dry cows, fresh cows, and lactating cows housed in separate pens. Before the experiment, we performed a power analysis via G*Power software (version 3.1.9.7; https://g-power.apponic.com) to achieve a statistical power (1 − β) of 0.80 and a type I error rate (α) of 0.05. The effect size for sample differences was estimated on the basis of previous studies (Poppy et al. 2012; Halfen et al. 2021; Guo et al. 2022). The analysis revealed that a sample size of 20 would be sufficient to achieve the desired statistical power for milk yield, blood biomarkers, and microbial analysis. To further increase the statistical power for milk yield analysis, we selected 100 cows per group. The experiment was conducted via a randomized complete block design with repeated measurements. Two hundred Holstein cows (Table 1) were enrolled in the study at dry-off, 60 d before the expected calving date, and they were divided into two groups on the basis of parity, BCS, and previous milk yield, and then randomly assigned within blocks to one of two treatments. The cows were allocated to the following treatment groups, with 100 cows in each group: no supplementation (CON) and supplementation with 19 g/d of SCFP per head (SCFP). The SCFP supplementation period spanned from d −60 to 60 d relative to the expected calving date. The SCFP, a fully fermented yeast culture containing residual yeast cells, fermentation metabolites, and growth media, was sourced from Diamond V (NutriTek^®^, Cedar Rapids, Iowa, USA). All diets were provided as a total mixed ration (TMR) (Table 2). The supplements were top-dressed onto the TMR once daily. After the TMR was delivered and the headlocks secured, a small divot was made on top of the TMR pile in front of each cow during the morning feeding. The SCFP supplements were then carefully placed into the divot. Cows were fed the far-off diet from −60 to −21 d before the expected calving date twice daily at approximately 0700 and 1400 h. From −21 d to parturition, cows received a close-up diet twice daily at the same time. From calving to 21 d postpartum, all the cows were fed a fresh diet three times daily at 0700, 1400, and 2100 h. From 21 to 140 d postpartum, the cows received a lactation diet three times daily at the same time. SCFP supplementation was discontinued from d 61 to investigate its carryover effect on milk production until d 140. The animals in this study were housed in free-stall barns, with far-off dry cows, close-up dry cows, fresh cows, and lactating cows kept in separate pens. All pens featured sand-bedded free-stall cubicles, with head-to-head cubicles measuring approximately 2.6 meters in length. The barns are naturally ventilated and supplemented with fans as needed, and lighting is provided by both natural and artificial sources as needed. Far-off and close-up dry cows were housed in pens with an additional exercise area to allow free movement. Cows were transferred from the far-off dry cow pen to the close-up pen 21 d before their expected calving date, where they remained until calving. After calving, they were transferred to a pen with cubicles as the resting area for early lactating cows until 21 d postpartum. Fresh water was provided ad libitum in each pen. Feeds were monitored at least 20 times daily to push up feed closer to the bunk to ensure access to feed at all times. A theoretical timeline illustrating the administration of treatments and data collection is depicted in Figure 1. The cows in each group were fed as a whole, and feed intake was not recorded.

**Table 1:** Parity, BCS, BW, and 305-d milk yield of dairy cows at enrollment (d −60 ± 6 relative to parturition).

Variable	**Treatment** ^1^	
	CON (Mean ± SD)	SCFP (Mean ± SD)
***N***	100	100
**Parity**	2.73 ± 0.76	2.70 ± 0.84
**BCS**	3.32 ± 0.46	3.38 ± 0.45
**BW, kg**	732.40 ± 87.66	729.80 ± 76.89
**305-d milk yield, kg**	9 570.79 ± 2 062.68	9 646.00 ± 2 141.70
**Subset^2^**		
***n***	20	20
**Parity**	2.75 ± 0.87	2.70 ± 0.61
**BCS**	3.43 ± 0.42	3.31 ± 0.38
**BW, kg**	729.90 ± 77.60	723.20 ± 49.74
**305-d milk yield, kg**	9 590.50 ± 2 181.27	9 607.38 ± 1 970.42

1 CON = 0 g/d *Saccharomyces cerevisiae* fermentation product (SCFP); SCFP = 19 g/d SCFP.

2 A subset of 40 cows was used for sample measurements (blood, ruminal, fecal and milk samples).

**Table 2:** Ingredients and chemical compositions of the experimental diets.

Items	Diet			
	Far off dry (d −60 to −21)	Close up dry (d −21 to 0)	Fresh (d 0 to 21)	Lactation (d 21 to 140)
**Ingredient, % of DM**				
** Oat hay**	22.30	17.67	2.18	—
** Barley**	8.92	—	—	—
** Alfalfa hay**	—	—	6.24	—
** Corn grain**	—	4.11	4.69	—
** Corn silage**	53.50	63.70	53.11	6.33
** Alfalfa silage**	—	—	—	48.96
** Soybean meal**	1.88	5.94	4.74	8.77
** Whole cottonseed**	—	—	—	4.61
** Cottonseed meal**	4.01	3.29	3.13	1.23
** Canola meal**	4.45	—	—	1.83
** Steam-flaked corn**	—	—	5.62	—
** Molasses**	—	—	3.13	5.31
** Beet pulp**	—	—	2.50	2.65
** Distillers dried grains with solubles (DDGS)**	3.57	2.05	—	1.43
** Distiller’s grains**	—	—	9.37	1.43
** Fat powder^1^**	—	—	0.47	13.26
** Fat acids calcium**	—	—	0.31	0.98
** Ruminal protected nicotinic acid**	—	—	0.07	0.31
** Choline chloride**	—	0.20	—	—
** Bypass soybean meal**	—	—	1.88	—
** Demolding agent**	0.04	0.02	—	1.43
** Saleratus**	—	—	0.31	—
** Limestone**	—	0.53	0.31	0.21
** Methionine^2^**	—	0.04	0.07	—
** Mineral and vitamin mix^3^^,^^4^**	1.33	2.45	1.87	1.26
**Chemical composition, % of DM**				
** DM**	45.06	44.83	46.10	47.28
** CP**	15.41	15.96	16.64	17.17
** NDF**	47.88	34.76	32.71	31.05
** ADF**	28.34	22.12	17.14	16.59
** Starch**	12.16	20.75	25.95	29.38
** EE**	2.55	2.72	4.27	4.64
** Ash**	9.75	8.97	8.49	7.83

1 Energy Booster 100 (MSC).

2 The guaranteed value of 2-hydroxy-4-methylthiobutyrate isopropyl is not lower than 57% (Adisseo).

3 Far off dry and close-up dry premix chemical composition of the premix contained (DM basis) 480 mg/kg Cu, 24 mg/kg I, 1,800 mg/kg Mn, 1,800 mg/kg Zn, 20 mg/kg Se, 24 mg/kg Co, 490,000 IU/kg vitamin A, 126,000 IU/kg vitamin D, and 2,100 mg/kg vitamin E.

4 Fresh premix and lactating premix chemical composition of the premix contained (DM basis) 480 mg/kg Cu, 28 mg/kg I, 1,600 mg/kg Mn, 1,800 mg/kg Zn, 13 mg/kg Se, 28 mg/kg Co, 210,000 IU/kg vitamin A, 70,000 IU/kg vitamin D, and 5,600 mg/kg vitamin E.

### Sampling, measurement, and analyses

*Feed samples*

TMR samples were collected weekly and stored at −20 °C until further processing. At the completion of the experiment, the samples were analyzed via wet chemistry methods following the Association of Official Analytical Chemists (AOAC 1995) for DM (method 930.15 of AOAC), CP (method 988.05 of AOAC), ether extract (EE, method 920.39 of AOAC), starch (analyzed via a total starch assay kit, method 996.11 of AOAC), and ash (method 924.05 of AOAC), NDF and ADF were analyzed via an ANKOM fiber analyzer (A2000i; American ANKOM, Macedon, New York, American) (Van Soest et al. 1991).

*Milk yield and components*

Milk yield was measured from all cows (*n* = 100 cows per treatment), and milk samples were collected from a subset of cows (*n* = 20 per treatment) selected on the basis of parity, BCS, and previous milk yield. The cows were milked three times a day at 0700, 1400, and 2100 h, and the milk yield was recorded at each milking from d 0–140 relative to parturition via software (Delaval TIANJIN Co., Ltd., Tianjin, China). Three consecutive milk samples (50 mL each) were collected on d 0 (the day of calving, approximately 6 h postpartum), 7, 14, 21, 28, 42, and 60 relative to parturition. The samples were pooled at a volume ratio of 4:3:3 on the basis of the morning, afternoon, and evening milking sessions. The samples were then preserved with potassium dichromate and stored at 4 °C. The concentrations of milk fat, protein, lactose, and total solids were analyzed by the Dairy Herd Improvement (DHI) Lab (DHI Laboratory of Gansu State Farms Tianmu Dairy Co., Ltd., Xcalibur 360EX, BOUMATIC) of the farm within 1 day after sampling. Weekly yields of milk components were calculated, as well as yields of 4% FCM: 4% fat correction milk = ([0.4 × kg of milk] + [15 × kg of milk fat]) and ECM: Energy correction milk = ([0.327 × kg of milk] + [12.95 × kg of milk fat] + [7.20 × kg of milk protein]). The colostrum was collected within 2 hr after parturition, and the colostrum yield was recorded. The quality was immediately assessed via the Brix scale (ATAGO, Shanghai, China).

*BCS and BW*

BCS and BW were measured from 40 cows (the same subset used for milk samples) on d −60 (actual date: −62 ± 3), −21 (actual date: −23 ± 3), 0, 21 and 60 relative to parturition. BCS was assessed by a trained evaluator.

*Blood samples*

Blood samples were collected from the same 40 cows on d −60, −21, −7 (actual date: −9 ± 3), 0, 3, 7, 21, and 60 relative to parturition. All blood samples were collected via the coccygeal vein before the morning feeding. Blood samples were collected in two 5-mL Vacutainer tubes containing no anticoagulant. The samples were left at room temperature for 2 h and centrifuged at 3,500 × *g* at 4 °C for 20 min. The samples were stored at −80 °C until analysis. The samples were analyzed for β-hydroxybutyrate (BHBA), total cholesterol (TC), glucose (GLU), non-esterified fatty acids (NEFA), total protein (TP), albumin (ALB), urea, total bilirubin (TBIL), IGF-1, total antioxidant capacity (T-AOC), superoxide dismutase (SOD), glutathione peroxidase (GSH-Px), malondialdehyde (MDA), IL-1β, tumor necrosis factor-alpha (TNFα), IL-6, serum amyloid A (SAA), lipopolysaccharide binding protein (LBP), and haptoglobin (HPT). All serum parameters were determined using commercial kits from Nanjing Jiancheng Bioengineering Institute and measured via an automatic biochemical analyzer (Hitachi 7600, Hitachi High-Technologies Corporation, Tokyo, Japan), all following the respective manufacturers’ instructions and operating protocols. The IL-1β cytokine assay was validated through linearity and spike-and-recovery experiments, with detailed results provided in Table S1.

*Ruminal fluids*

Ruminal fluid samples were obtained from the same subset of 40 cows on d −60, 0, 21, and 60 relative to parturition. The samples were collected via an oral stomach tube (with a 2 mm wall thickness and a 6 mm internal diameter; Anscitech Co., Ltd., Wuhan, China) approximately 4 h after the morning feeding, without restricting access to feed. Ruminal fluid samples were immediately measured for ruminal pH with a glass electrode pH meter (BELL Analytical Instruments Co., Ltd., Dalian, China). Ruminal fluid fractions were obtained by filtering ruminal fluid through four layers of cheesecloth. The samples were stored at −20 °C for subsequent analysis of volatile fatty acid (VFA) and ammonia N (NH_3_-N) concentrations.

The supernatant of the ruminal fluid samples was used to analyze NH_3_-N, following the assay described by Broderick and Kang (Broderick and Kang 1980). VFA concentrations were measured via an automated gas chromatograph (model 689, HewlettPackard, American) equipped with a 0.25-mm internal diameter × 15-m capillary column (Nukol 24106-U, Sulpeco Inc.), with 2-ethylbutyrate as the internal standard (Erwin et al. 1961).

The ruminal fluids (10 mL each) collected on d 60 were snap frozen in liquid nitrogen and then stored at −80 °C for subsequent analysis of microbial composition and population via 16S rDNA sequencing according to the method described by Li et al. (Li et al. 2019).

*Fecal samples*

The total apparent tract digestibility was measured on d 60. Fecal grab samples were collected every 12 h for 3 d from the same 40 cows on d 59, 60, and 61 relative to parturition and frozen at −20 °C. These samples were pooled from cows and analyzed for acid insoluble ash (AIA) as an internal marker to calculate the apparent digestibility of DM, ADF, NDF, CP, and EE (Van Keulen and Young 1977).

### DNA extraction and sequencing

The bacterial genomic DNA was isolated from ruminal fluid via the MagAttract PowerSoil Pro DNA Kit manual (Qiagen Inc., Germany). The quantification and quality check of the extracted DNA were performed with a NanoDrop 2000 spectrophotometer (Thermo Fisher Scientific Inc., American), and the DNA was stored at −80 °C until further use. The V3-V4 region of the bacterial 16S rRNA gene was amplified with primer pairs (338F: 5′-ACTCCTACGGGAGGCAGCAG-3′, 806R: 5′-GGACTACHVGGGTWTCTAAT-3′) via an ABI GeneAmp 9700 PCR thermocycler (ABI, California, USA) (Liu et al. 2016). The thermocycling conditions for PCR involved a 3 min initial denaturation step at 95 °C, followed by 20 cycles, including 10 s of denaturation at 98 °C, 10 s of annealing at 59 °C, 45 s of extension at 72 °C, and a 2 min final extension step at 72 °C. All the samples were amplified in triplicate. The PCR product was extracted from a 2% agarose gel and purified via the AxyPrep DNA Gel Extraction Kit (Axygen Biosciences, Union City, California, USA) according to the manufacturer’s instructions and quantified via a Quantus Fluorometer (Promega, USA). The purified amplicons were pooled in equimolar amounts and sequenced on an Illumina MiSeq PE300 platform (Illumina, San Diego, USA) according to the standard protocols of Majorbio Bio-Pharm Technology Co. Ltd. (Shanghai, China). The raw sequencing reads were deposited into the NCBI Sequence Read Archive database.

After demultiplexing, the resulting sequences were quality filtered with Fastp (Chen et al. 2018) (https://github.com/OpenGene/fastp, version 0.19.6) and merged with FLASH (Magoč and Salzberg 2011) (http://www.cbcb.umd.edu/software/flash, version 1.2.11). The QIIME2 pipeline (Bolyen et al. 2019) was used with default parameters to apply the DADA2 (Callahan et al. 2016) plugin for denoising the optimized sequences resulting from quality control and read merging. These sequences were commonly referred to as amplicon sequence variants (ASVs) after DADA2 denoising. On the basis of the Silva 16S rRNA gene database (v 138), the naive Bayes classifier in QIIME 2 was used to perform species-level classification analysis of the ASVs. The spike-in ASVs were identified and extracted, and standard curve equations were constructed on the basis of the number of spike-in ASV sequencing sequences in each sample. The absolute copy number of each taxonomic group in each sample was calculated, and the 16S copy number was corrected via the rrnDB database (https://rrndb.umms.med.umich.edu/) (Stoddard et al. 2015).

### Calculations and statistical analysis

Before analysis, the normality of residuals and homogeneity of variance for each variable analyzed were examined via the Shapiro–Wilk test. The data are presented in tables and graphs on the original scale (mean and SEM). Data were analyzed as repeated measures with the MIXED procedure of SAS version 9.4 (SAS Institute Inc.), incorporating treatment, time (day or week), and two-way interactions as fixed effects. Various covariance structures, including AR (1), ANTE (1), CS, Simple and UN, were examined to find the best-fit structure for the model on the basis of the lowest Akaike information criterion. Differences among treatments were considered significant at *P *< 0.05, and trends toward significance were declared at 0.05 ≤ *P *< 0.10.

On the basis of the ASV information, rarefaction curves and alpha diversity indices, including observed ASVs, Chao1 richness, the Shannon index, and Good’s coverage, were calculated with Mothur v1.30.1 (Schloss et al. 2009). The similarity among the microbial communities in different samples was determined via principal coordinate analysis (PCoA) on the basis of Bray–Curtis dissimilarity via the Vegan v2.5-3 package. The PERMANOVA test was used to assess the percentage of variation explained by the treatment along with its statistical significance via the Vegan v2.5-3 package. Linear discriminant analysis (LDA) effect size (LEfSe) (http://huttenhower.sph.harvard.edu/LEfSe) was performed to identify the significantly abundant taxa of bacteria among the different groups (LDA score > 2.5, *P *< 0.05) (Segata et al., 2011). To address multicollinearity among clinical parameters, the variance inflation factor (VIF) for each variable was estimated via the VIF function in the car package (https://cran.r-project.org/web/packages/car/car.pdf).

## Results

### Milk production

**Figure 2:**
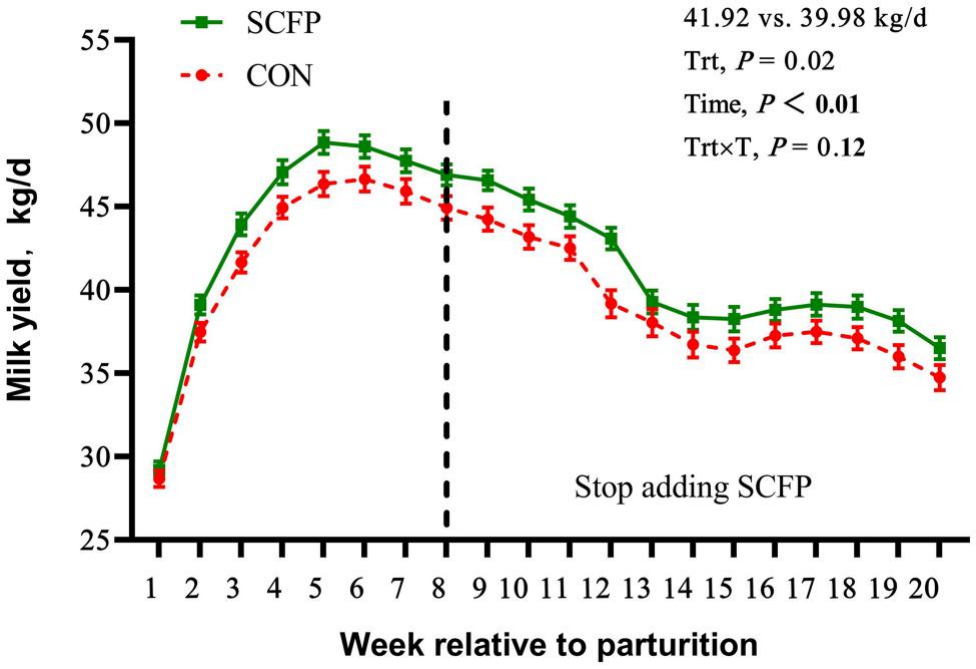
Milk production during the first 20 wk relative to parturition for cows supplemented with SCFP (*n* = 100). Trt × T = interaction of treatment × time. Values are means, and SE are represented by vertical bars. The cows were housed in the fresh pen and fed fresh diet before 3 wk and then moved to the high-producing pen and fed lactation diet after 3 wk. Between weeks 12 and 15, a transition in farm management led to fluctuations in the overall milk production of the herd.

The average daily milk yield was greater in SCFP cows than in CON cows at 60 d following calving (43.93 vs. 42.08 kg/d; *P *= 0.03; *n* = 100; Table 3). This trend continued over the first 20 wk postpartum even after SCFP supplementation was stopped, with SCFP cows producing more milk on average than CON cows did (41.92 vs. 39.98 kg/d; *P *= 0.04; *n* = 100; Figure 2). 3.5% FCM and ECM were not affected by the dietary treatments (Table 3). SCFP supplementation also increased lactose yield (2.24 vs. 2.09 kg/d, *P *= 0.04; Table 3). The percentages and yields of milk fat, protein, and solids were not significantly affected by the dietary treatments during the 60 d following calving (Table 3). The colostrum yield and Brix were not affected by the dietary treatments (Table 3).

**Table 3:** Effects of supplementation with *Saccharomyces cerevisiae* fermentation product (SCFP) on milk production parameters from d 0 to 60^1^.

Items	**Treatmen**t^2^			***P*-value** ^3^		
	CON	SCFP	SEM	Trt	Time	Trt × T
**Milk yield, kg/d (*n* = 100)**	42.08	43.93	0.83	0.03	<0.01	0.06
**Milk yield, kg/d (*n* = 20)**	39.75	42.03	1.07	0.04	<0.01	0.23
**ECM^4^, kg/d (*n* = 20)**	47.48	50.49	1.58	0.18	<0.01	0.98
**4% FCM^5^, kg/d (*n* = 20)**	44.31	47.48	1.59	0.17	0.01	0.97
**Milk composition (*n* = 20)**						
** Fat, %**	4.29	4.33	0.12	0.80	<0.01	0.06
** Fat, kg/d**	1.89	2.02	0.09	0.32	0.08	0.92
** Protein, %**	3.52	3.48	0.04	0.41	<0.01	0.29
** Protein, kg/d**	1.38	1.43	0.04	0.41	<0.01	0.05
** Lactose, %**	5.29	5.20	0.03	0.54	<0.01	0.90
** Lactose, kg/d**	2.09	2.24	0.05	0.04	<0.01	0.08
** Solids, %**	15.95	15.75	0.24	0.55	<0.01	0.94
** Solids, kg/d**	6.29	6.67	0.18	0.15	<0.01	0.98
** Colostrum yield, L**	4.54	4.58	0.29	0.52	—	—
** Colostrum Brix, %**	25.53	24.72	2.49	0.31	—	—

1 Milk samples were collected on d 7, 14, 21, 28, 42 and 60 relative to parturition.

2 CON = 0 g/d SCFP; SCFP = 19 g/d SCFP; a subset of 40 cows was used for sample measurements.

3 Trt × T = interaction of treatment × time (week).

4 ECM: Energy correction milk = [(0.327 × kg of milk) + (12.95 × kg of milk fat) + (7.20 × kg of milk protein)].

5 4% FCM: 4% fat correction milk = ([0.4 × kg of milk] + [15 × kg of milk fat]).

### BW, BCS and apparent digestibility

As shown in Table 4, no significant differences in BW (698.96 vs. 712.79 kg; *P *= 0.45) or BCS (3.19 vs. 3.17; *P *= 0.53) from −60 to 60 d relative to parturition were detected between cows fed the SCFP and CON diets. Compared with the CON group, the SCFP group exhibited a smaller decline in BCS during the fresh period (d 0 to 21) (*P *= 0.03). The apparent digestibility of NDF (*P *= 0.06) and ADF (*P *= 0.07) tended to increase in the SCFP group compared with the CON group (Table 5).

**Table 4:** Effects of supplementation with *Saccharomyces cerevisiae* fermentation product (SCFP) on BCS and BW^1^.

Items	**Treatment** ^2^			***P*-value** ^3^		
	CON	SCFP	SEM	Trt	Time	Trt × T
***n***	20	20				
**BCS^4^**	3.17	3.19	0.02	0.53	<0.01	0.49
**d −60 to −21 change**	0.06	0.01	0.02	0.34	—	—
**d −21 to 0 change**	**−**0.15	**−**0.22	0.02	0.06	—	—
**d 0 to 21 change**	**−**0.22	**−**0.15	0.02	0.03	—	—
**d 21 to 60 change**	**−**0.04	**−**0.07	0.01	0.35	—	—
***n***	20	20				
**BW, kg**	712.79	698.96	12.76	0.45	<0.01	0.97
**d −60 to −21 change**	24.18	24.88	2.57	0.89	—	—
**d −21 to 0 change^5^**	**−**18.90	**−**22.98	3.37	0.55	—	—
**d 0 to 21 change**	**−**53.78	**−**51.55	6.04	0.86	—	—
**d 21 to 60 change**	**−**2.75	1.58	3.74	0.57	—	—

1 BCS and BW were measured on d **−**60, **−**21, 0, 21, and 60 relative to parturition.

2 CON = 0 g/d SCFP; SCFP = 19 g/d SCFP.

3 Trt × T = interaction of treatment × time (day).

4 BCS-related variables are measured in BCS units (1 to 5, where 1 is emaciated and 5 is obese).

5 BW on d 0 was measured after calving.

**Table 5:** Effects of supplementation with *Saccharomyces cerevisiae* fermentation product on apparent digestibility on d 60^1^.

Items^2^	Treatment^3^		SEM	*P*-value
	CON	SCFP		
***N***	20	20		
**DM, %**	72.46	73.39	0.42	0.16
**CP, %**	70.90	70.82	0.84	0.96
**NDF, %**	56.88	59.75	0.77	0.06
**ADF, %**	52.68	55.58	0.87	0.07
**EE, %**	65.23	66.97	1.36	0.13

1 The digestibility was measured on d 60 relative to parturition.

2 DM, dry matter; CP, crude protein; NDF, neutral detergent fiber; ADF, acid detergent fiber; EE, ether extract.

3 CON = 0 g/d SCFP; SCFP = 19 g/d SCFP.

### Ruminal fermentation parameters

**Figure 3:**
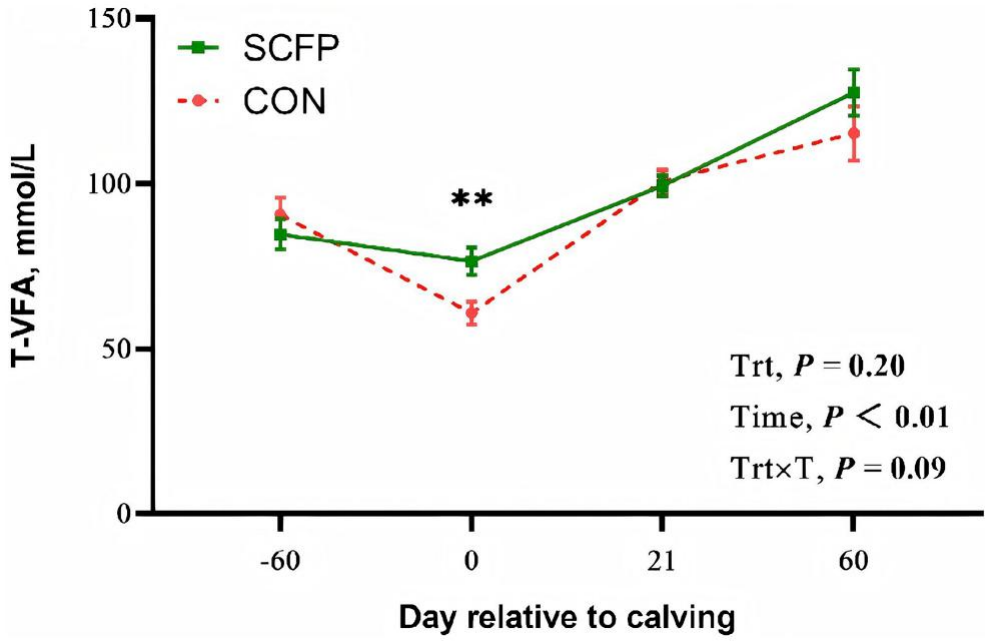
Ruminal fermentation of T-VFA of feeding CON diet or SCFP diet during the experimental period. Mean separations between diets at a given time point were evaluated, differences (*) were declared at *P *< 0.05, differences (**) were declared at *P *< 0.01. Values are means, and SE are represented by vertical bars.

The addition of SCFP had no significant effect on ruminal pH, NH_3_-N, or VFA concentrations in dairy cows (Table 6). However, trends of interaction between treatment and time were observed for the concentrations of total VFAs (*P *= 0.09; Table 6). On d 0 (the day of calving), the concentrations of total VFAs were greater in the group fed SCFP than in the CON group (*P *< 0.01; Figure 3A).

**Table 6:** The effects of supplementation with *Saccharomyces cerevisiae* fermentation product (SCFP) on ruminal fermentation^1^.

Items^2^	**Treatment** ^3^			***P*-value** ^4^		
	CON	SCFP	SEM	Trt	Time	Trt × T
***n***	20	20				
**pH**	6.59	6.53	0.05	0.39	<0.01	0.45
**NH_3_-N, mmol/L**	7.94	8.31	0.26	0.32	<0.01	0.24
**T-VFA, mmol/L**	91.84	97.03	2.83	0.20	<0.01	0.09
**VFA, mol/100 mol of T-VFA**						
** Acetate**	52.54	52.87	0.35	0.44	<0.01	0.67
** Propionate**	27.71	27.50	0.30	0.59	<0.01	0.63
** Isobutyrate**	1.21	1.15	0.04	0.29	<0.01	0.64
** Butyrate**	14.64	14.71	0.18	0.78	<0.01	0.44
** Isovalerate**	1.91	1.90	0.05	0.85	<0.01	0.87
** Valerate**	1.99	1.93	0.05	0.43	<0.01	0.63
** A/P**	1.98	2.01	0.03	0.54	<0.01	0.69

1 Ruminal fermentation was measured on d **−**60, 0, 21, and 60 relative to parturition.

2 NH_3_-N, urea nitrogen; T-VFA, total volatile fatty acid; A/P, ratio of acetic acid concentration to propionic acid concentration.

3 CON = 0 g/d SCFP; SCFP = 19 g/d SCFP.

4 Trt × T = interaction of treatment × time (day).

### Blood parameters

The main effects and interactions for blood biomarkers of metabolites, oxidative status parameters, and inflammatory markers were presented in Table 7. Compared with the CON group, cows fed with SCFP exhibited higher concentrations of T-AOC (*P *= 0.02), SOD, and GSH-Px (*P *< 0.01), and lower concentrations of BHBA (*P *= 0.04), NEFA (*P *< 0.01), TBIL (*P *= 0.04), MDA (*P *< 0.01), IL-1β (*P *< 0.01), TNF-α (*P *< 0.01), SAA (*P *< 0.01), LBP (*P *= 0.01), and HPT (*P *= 0.03).

**Table 7:** Effects of supplementation with *Saccharomyces cerevisiae* fermentation product (SCFP) on blood biomarkers^1^.

Items^2^	**Treatment** ^3^			*P*-values^4^		
	CON	SCFP	SEM	Trt	Time	Trt × T
***n***	20	20				
**Serum metabolites**						
** BHBA, μmol/L**	552.80	517.14	11.62	0.04	< 0.01	0.02
** TC, mmol/L**	3.10	3.23	0.11	0.45	< 0.01	0.55
** GLU, mmol/L**	3.91	3.89	0.09	0.90	< 0.01	0.91
** NEFA, mmol/L**	0.36	0.33	0.01	< 0.01	< 0.01	0.03
** TP, g/L**	71.24	71.59	0.89	0.82	< 0.01	0.15
** ALB, g/L**	32.59	33.12	0.29	0.20	< 0.01	0.43
** Urea, mmol/L**	5.26	5.34	0.13	0.67	< 0.01	0.99
** TBIL, μmol/L**	4.43	3.67	0.26	0.04	< 0.01	0.60
** IGF-1, pg/mL**	141.32	147.14	2.83	0.15	< 0.01	0.02
**Oxidative status parameters**						
** T-AOC, μmol/L**	0.24	0.25	0.003	0.02	< 0.01	< 0.01
** SOD, U/mL**	156.62	163.67	1.29	< 0.01	< 0.01	< 0.01
** GSH-Px, U/mL**	173.41	186.08	2.29	< 0.01	< 0.01	0.04
** MDA, nmol/mL**	3.06	2.48	0.87	< 0.01	0.07	0.18
**Inflammatory markers**						
** IL-1β, pg/mL**	34.70	27.35	1.54	< 0.01	< 0.01	< 0.01
** TNF-α, pg/mL**	88.92	72.99	3.34	< 0.01	0.23	< 0.01
** IL-6, pg/mL**	73.24	65.94	5.11	0.32	< 0.01	0.91
** SAA, μg/mL**	198.59	187.66	0.83	< 0.01	< 0.01	< 0.01
** LBP, μg/mL**	148.04	127.43	5.62	0.01	< 0.01	0.33
** HPT, μg/mL**	317.28	286.42	9.81	0.03	< 0.01	0.04

1 Blood biomarkers were measured on d −60, −21, −7, 0, 3, 7, 21, and 60 relative to parturition.

2 BHBA, β-hydroxybutyrate; TC, total cholesterol; GLU, glucose; NEFA, non-esterified fatty acids; TP, total protein; ALB, albumin; TBIL, total bilirubin; T-AOC, total antioxidant capacity; SOD, superoxide dismutase; GSH-Px, glutathione peroxidase; MDA, malondialdehyde; TNFα, tumor necrosis factor-alpha; SAA, serum amyloid A; LBP, lipopolysaccharide binding protein; and HPT, haptoglobin.

3 CON = 0 g/d SCFP; SCFP = 19 g/d SCFP.

4 Trt × T = interaction of treatment × time (day).

**Figure 4:**
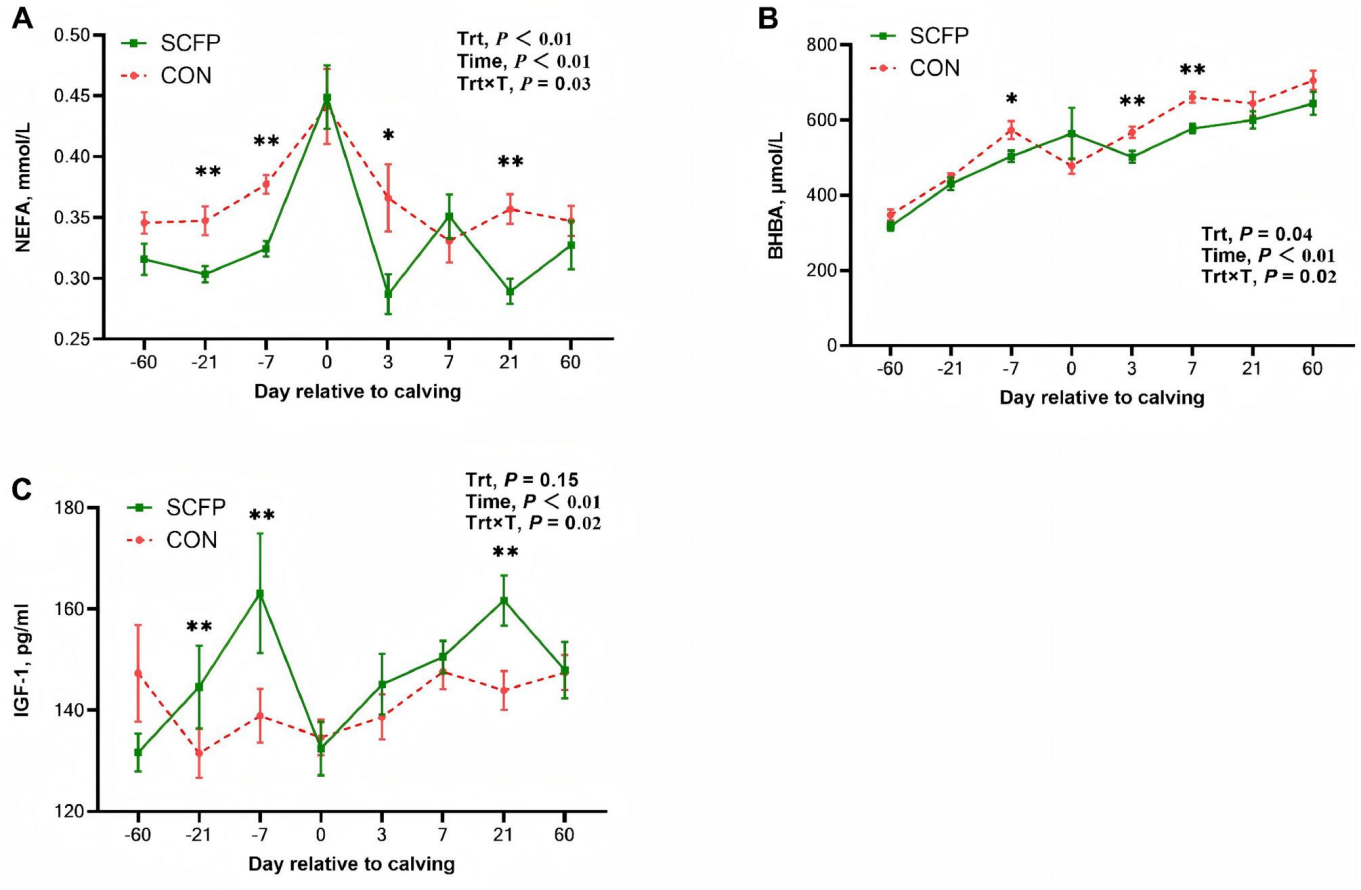
Serum metabolites of NEFA (A), BHBA (B), and IGF-1 (C) of feeding CON diet or SCFP diet during the experimental period. Mean separations between diets at a given time point were evaluated, differences (*) were declared at *P *< 0.05 and (**) were declared at *P *< 0.01. Values are means, and SE are represented by vertical bars.

NEFA concentrations were significantly lower in the SCFP group at d −21 (*P *< 0.01), −7 (*P *< 0.01), 3 (*P *< 0.05), and 21 (*P *< 0.01) (Figure 4A). BHBA concentrations were significantly lower in the SCFP group at d −7 (*P *< 0.05), 3 (*P *< 0.01), and 7 (*P *< 0.01) (Figure 4B). The IGF-1 concentrations were significantly greater in the SCFP group than in the control group at d −21, −7, and 21 (*P *< 0.01; Figure 4C).

**Figure 5:**
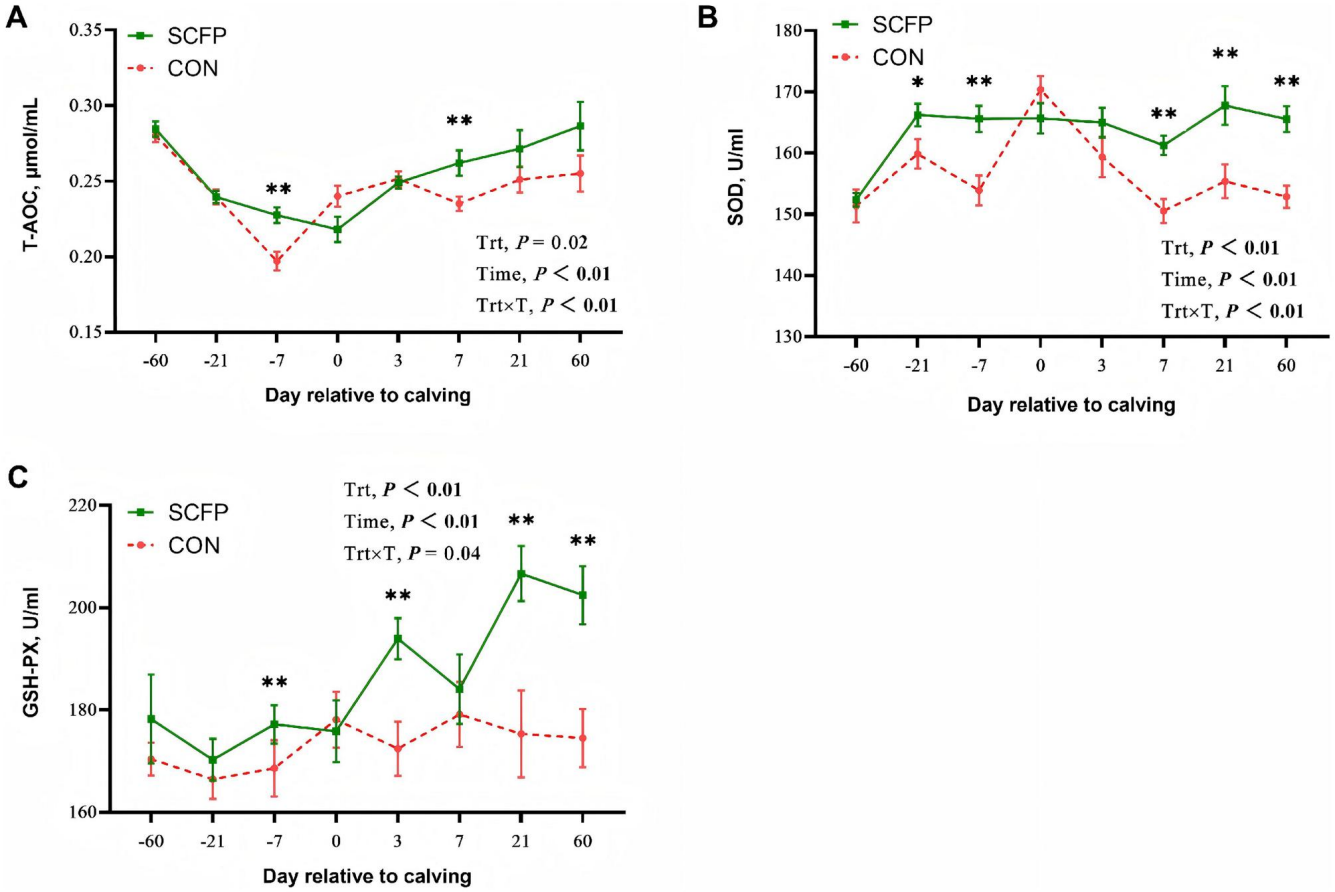
Oxidative status parameters of T-AOC (A), SOD (B), and GSH-Px (C), of feeding CON diet or SCFP diet during the experimental period. Mean separations between diets at a given time point were evaluated, differences (*) were declared at *P *< 0.05 and (**) were declared at *P *< 0.01. Values are means, and SE are represented by vertical bars.

T-AOC concentrations were significantly greater in the SCFP group at d −7 and 7 (*P *< 0.01) (Figure 5A). SOD concentrations were significantly greater in the SCFP group at d −21 (*P *< 0.05), −7 (*P *< 0.01), 7 (*P *< 0.01), 21 (*P *< 0.01), and 60 (*P *< 0.01) (Figure 5B). The GSH-Px concentrations were significantly greater in the SCFP group than in the control group at d −7, 3, 21, and 60 (*P *< 0.01; Figure 5C).

**Figure 6:**
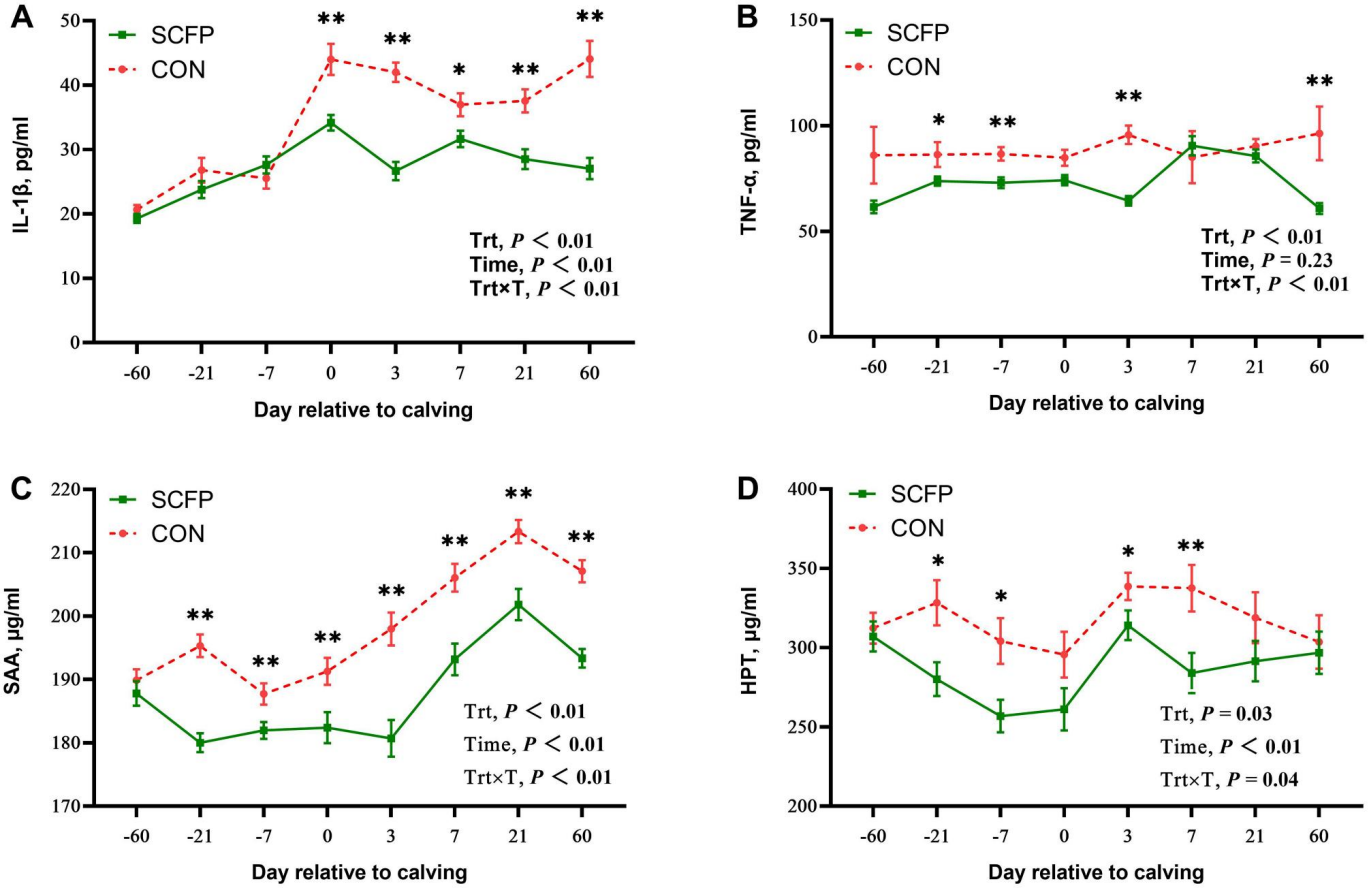
Inflammatory markers of IL-1β (A), TNF-α (B), SAA (C), and HPT (D) of feeding CON diet or SCFP diet during the experimental period. Mean separations between diets at a given time point were evaluated, differences (*) were declared at *P *< 0.05 and (**) were declared at *P *< 0.01. Values are means, and SE are represented by vertical bars.

IL-1β concentrations were significantly lower in the SCFP group at d 0 (*P *< 0.01), 3 (*P *< 0.01), 7 (*P *< 0.05), 21 (*P *< 0.01), and 60 (*P *< 0.01) (Figure 6A). TNF-α concentrations were significantly lower in the SCFP group at d −21 (*P *< 0.05), −7 (*P *< 0.01), 3 (*P *< 0.01), and 60 (*P *< 0.01) (Figure 6B). The SAA concentrations were significantly lower in the SCFP group than in the control group at d −21, −7, 0, 3, 7, 21, and 60 (*P *< 0.01; Figure 6C). HPT concentrations were significantly lower in the SCFP group at d −21 (*P *< 0.05), −7 (*P *< 0.05), 3 (*P *< 0.05), and 7 (*P *< 0.05) (Figure 6D).

### Ruminal bacterial diversity and community structure

**Figure 7:**
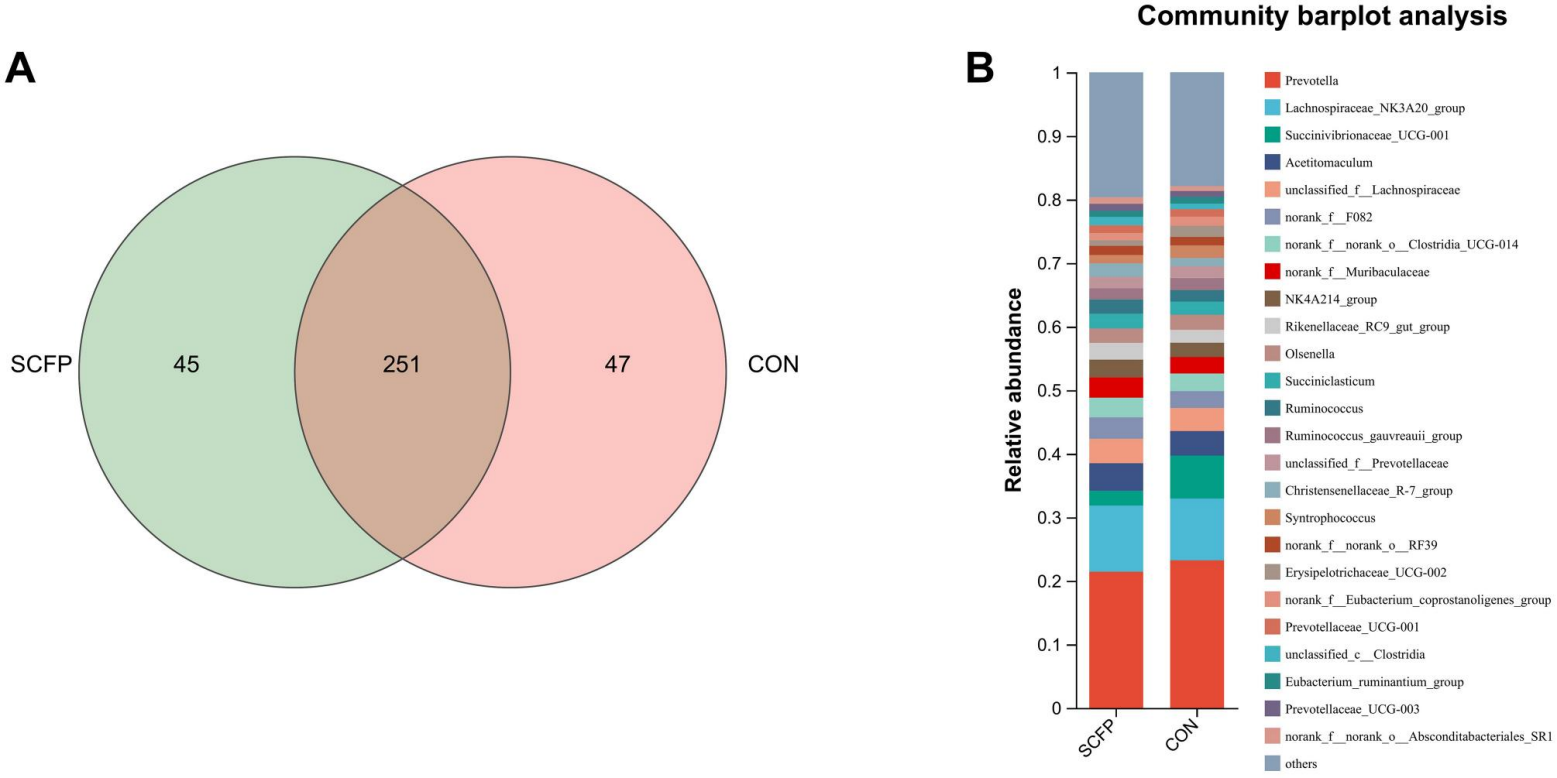
Effects of SCFP administration on ruminal microbiota at the genus level. A) Venn Diagram of shared genera between the two groups. B) The composition of ruminal microbiota at the genus level in the CON and SCFP group.

Amplicon sequencing of the 16S rDNA yielded a total of 343 genera from 40 ruminal fluid samples at the genus level. Among these samples, 251 genera were shared between the groups, with 45 genera exclusively found in the SCFP group and 47 genera exclusive to the CON group (Figure 7A). The dominant bacteria identified in the ruminal fluid were *Prevotella, Lachnospiraceae_NK3A20_group, unclassified_f_Lachnospiraceae, Succinivibrionaceae_UCG-001, Acetitomaculum*, and *norank_f_F082* (Figure 7B).

**Figure 8:**
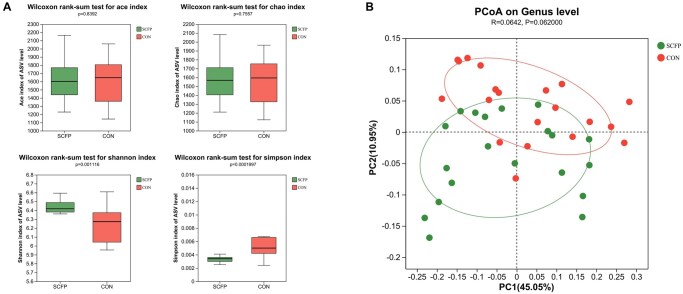
Effects of SCFP administration on ruminal microbiota diversity of cows on d 60 relative to parturition. A) Alpha-diversity in ruminal fluid. B) Beta-diversity in ruminal fluid.

The indices of α diversity showed that SCFP administration significantly increased the Shannon index (*P *< 0.01) and decreased the Simpson index (*P *< 0.01), but no significant differences were found in the ACE and Chao 1 indices in the ruminal fluid microbiota (Figure 8A). Principal coordinate analysis (PCoA) based on the Bray–Curtis distances revealed a tendency for separation between the bacterial communities in the two groups in the ruminal fluid (*P *= 0.06; Figure 8B).

### Different bacterial taxa

**Figure 9:**
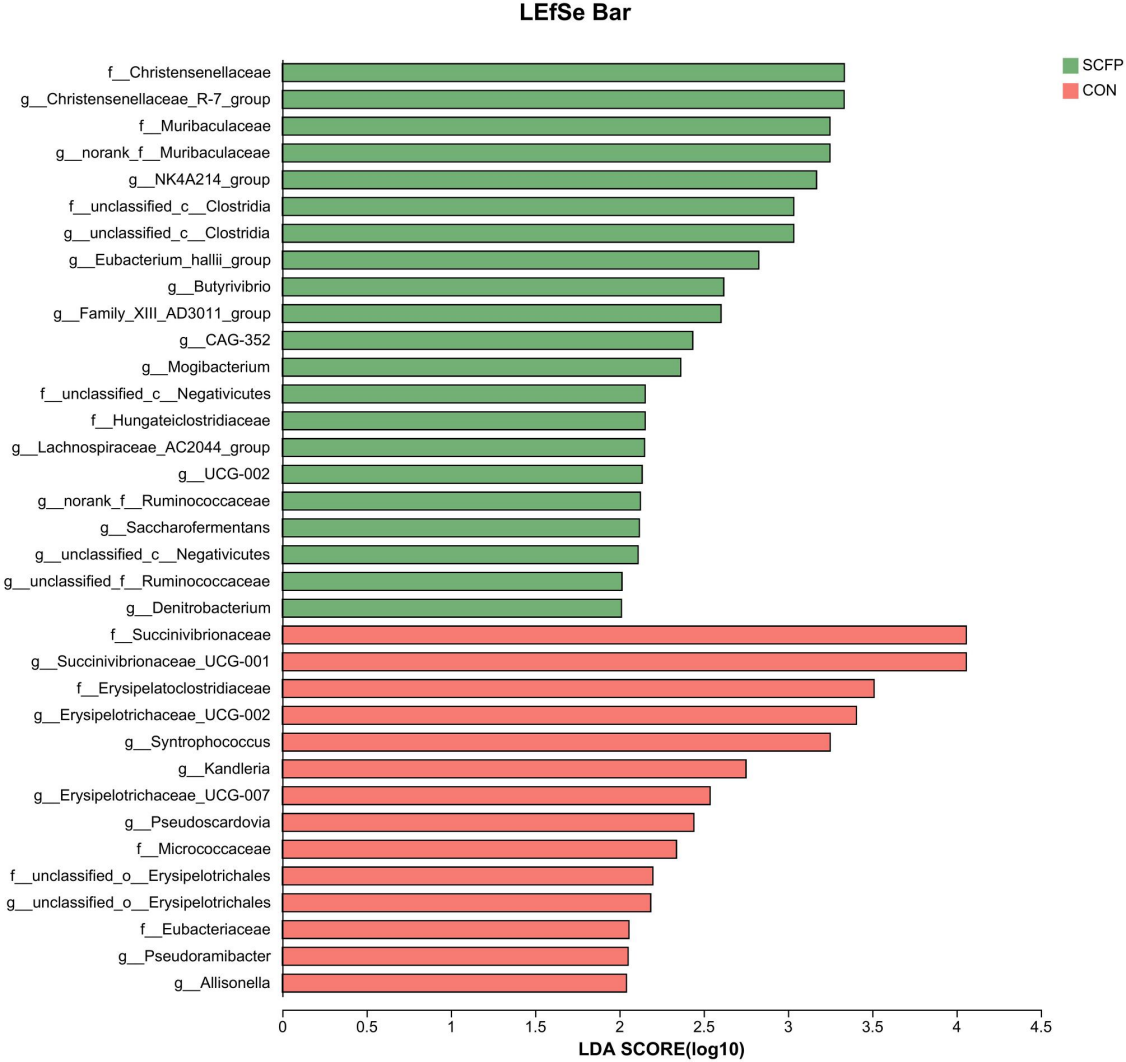
Differential microbiota at the genus level of ruminal fluid between SCFP and CON on d 60 relative to parturition.

LEfSe was conducted with LDA values of taxa greater than 2 to assess differential abundance at the genus level between the CON and SCFP groups (LDA > 2.5; *P *< 0.05; Figure 9). In the ruminal fluids, *Christensenellaceae_R-7_group, norank_f_Muribaculaceae, unclassified_c_Negativicutes, Denitrobacterium, norank_f_norank_o_norank_c_norank_p_WPS-2, unclassified_c_Clostridia, NK4A214_group, Eubacterium_hallii_group, Anaerofustis, UCG-009, Family_XIII_AD3011_group, Butyrivibrio, Mogibacterium, UCG-002, Anaerovibrio, Lachnospiraceae_FE2018_group, Papillibacter, CAG-352, Anaerovorax, Lachnospiraceae_AC2044_group, U29-B03, V9D2013_group, Saccharofermentans*, and *unclassified_f_Ruminococcaceae* presented significantly greater abundances in the SCFP group than in the CON group.

## Discussion

Limited research exploring the effects of dietary SCFP supplementation on dairy cows from the day of dry-off through early lactation is currently available. This study revealed that supplementing with SCFP from dry-off to early lactation affected milk yield, ruminal fermentation, blood parameters, and ruminal microbiota in dairy cows.

Microorganisms play crucial roles in animal health by significantly contributing to nutrient metabolism, promoting growth and development, and regulating the immune system (Cui et al. 2020). Cows supplemented with SCFP presented a significant increase in the abundance of *NK4A214_group* in rumen. *NK4A214_group* can also increase growth and lactation performance (Huang et al. 2021; Wang et al. 2023). *Christensenellaceae_R-7_group*, which is significantly enriched in the SCFP group belonging to the Christensenellaceae family, aids in the fermentation of structural carbohydrates (Morotomi et al. 2012) and increases milk production in dairy cows (Huang et al. 2021; Wang et al. 2023).

The addition of SCFP increases DM digestibility (Miller-Webster et al. 2002) and has been shown to improve fiber digestion (Dias et al. 2018). Our results revealed similar outcomes with increasing trends of apparent NDF and ADF digestibility, which may be related to the ability of SCFP to increase the abundance of carbohydrate-degrading microorganisms in the rumen. Studies related to ruminal VFAs have indicated that SCFP increases the production of ruminal propionate in dairy cows (Miller-Webster et al. 2002). Increased propionate enters the liver, providing intermediates for the tricarboxylic acid cycle, allowing the oxidation of acetyl-CoA and ultimately increasing ATP production (Allen et al. 2009). Our study indicated that although there were no differences in ruminal fermentation levels between the two treatment groups, SCFP exhibited a stronger promoting effect on ruminal fermentation in cows on the day of parturition.

Dairy cows from close-up to early lactation are prone to adipose mobilization, during which triglycerides are released into the bloodstream in the form of glycerol and NEFA (Drackley 1999). While NEFA serve as precursors for energy and milk fat synthesis in cows, elevated concentrations of NEFA can disrupt liver function and increase the risk of ketosis (Bobe et al. 2004). The concentrations of NEFA in the blood can be used as a marker for the degree of adipose mobilization in cows. BHBA is synthesized from incomplete oxidation of fatty acids, and elevated BHBA concentrations are detrimental to the immune function of the body, increasing the risk of disease in cows (Ingvartsen and Moyes 2013). Research has demonstrated significant reductions in NEFA and BHBA concentrations in the blood of cows after being fed SCFP (Zaworski et al. 2014; Dai et al. 2024). This study, due to the interval between blood sampling and calving being less than 1 hr on the day of calving, this excessively short interval caused a sharp increase in NEFA (Grummer 1995b). As negative energy balance alleviated, NEFA gradually stabilized. Meanwhile, postpartum BHBA remained at a high level, which may be associated with sampling stress, impairing the overall postpartum recovery of the herd. In ruminants, glucose production is primarily mediated by hepatic gluconeogenesis (Aschenbach et al. 2010), and enhancing the function of hepatic gluconeogenesis is also an effective strategy to improve milk yield in dairy cows (Karcher et al. 2007). This process can provide additional energy for dairy cows, which not only supports higher milk production but also effectively alleviates postpartum negative energy balance (Grummer 1995a). In the present study, although there were no significant differences in ruminal fermentation levels between the two groups, SCFP exhibited a stronger promoting effect on ruminal fermentation in dairy cows on the day of calving. Furthermore, despite no overall significant difference in blood glucose levels between the two groups, cows in the SCFP group experienced less body condition loss during the peripartum period and had lower concentrations of serum NEFA and BHBA. This phenomenon may be associated with the faster recovery of feed intake in the SCFP group—faster feed intake recovery not only provides sufficient substrates for hepatic gluconeogenesis, thereby enhancing gluconeogenic activity, but also simultaneously improves the energy metabolic status of dairy cows both pre- and postpartum. However, due to the group-housing model adopted in the current experiment, it is not possible to accurately determine the daily dry matter intake of each individual dairy cow. Therefore, subsequent studies need to further collect individual cow feed intake data to thoroughly investigate the impact of SCFP on feed intake and its associations with other indicators.

Changes in oxidative markers (GSH-Px, SOD, MDA, and TAOC) serve as indicators of the strength of the antioxidant capacity of cows, which can be used to assess their physiological and health status (Gaál et al. 2006). Glutathione (GSH) primarily acts as a reducing agent in redox reactions, while GSH-Px is considered an antioxidant enzyme that catalyzes the reduction of hydrogen peroxide in the presence of GSH; moreover, MDA is a product of lipid peroxidation induced by oxygen free radicals (Zhang et al. 2020). Animals can utilize exogenous antioxidants in conjunction with endogenous antioxidants to counteract the effects of reactive oxygen species (Miller et al. 1993). SCFP product containing yeast possesses significant antioxidant properties and are capable of scavenging free radicals within the body, reducing redox potential, and inhibiting the growth of strict anaerobic bacteria in the ruminal environment (Newbold et al. 1996). Additionally, in vitro cell experiments have shown that SCFP can provide protection to cells against oxidative damage (Jensen et al. 2008). In vivo studies have also shown that supplementing SCFP to transition dairy cows can support antioxidant effects (Dai et al. 2024). Consistent with previous research, our study indicated that supplementation with SCFP significantly increased the concentrations of T-AOC, SOD, and GSH-Px in the serum of cows compared with those in the CON group, whereas the MDA concentration significantly decreased. These findings suggest that SCFP significantly enhances the antioxidant capacity of cows. As cellular metabolic activity increases, the production of reactive oxygen species gradually increases, exceeding the neutralizing capacity of endogenous antioxidants and leading to oxidative stress in the body (Sivinski et al. 2022). This stress can result in tissue damage, immune suppression, increased disease susceptibility, and decreased productivity (Abuelo et al. 2015).

Cows experience a certain degree of inflammatory response during parturition; although individual variations exist, this inflammatory response is a common physiological state during the periparturient period (Bradford et al. 2015; Mezzetti et al. 2020). Additionally, the peak of lactation may also lead to immune dysfunction and decreased anti-inflammatory capacity in cows (Sordillo and Aitken 2009; Sordillo and Raphael 2013). Numerous studies have investigated the immunomodulatory effects of SCFP and confirmed positive immunomodulatory effects under heat stress and respiratory and mastitis challenge conditions (Al-Qaisi et al. 2020; Mahmoud et al. 2020; Vailati-Riboni et al. 2021). SCFP is known to contain various bioactive compounds, such as beta-glucans, B vitamins, nucleotides, and amino acids, which are associated with the activation of the immune system (Hristov et al. 2010), thereby interacting with the immune system of supplemented cows and regulating inflammation levels in the rumen (Trevisi et al. 2014; Trevisi and Minuti 2018) and intestines (Jawhara et al. 2012). The active components in SCFP can reduce bacterial adhesion and mitigate chronic inflammation caused by harmful bacteria (Li et al. 2016). Supplementation with SCFP during the transition period can reduce the HPT concentration in early lactation (Knoblock et al. 2019). SCFP supplementation also reduces the expression of the proinflammatory cytokine IL-1β, thereby lowering the risk of inflammation in transition dairy cows (Dai et al. 2024). Notably, our findings reveal that IL-1β levels remain persistently elevated throughout the postpartum period, which deviates from previously documented conventional outcomes (Alharthi et al. 2018; Dai et al. 2024). Given this discrepancy, follow-up investigations are warranted to further validate the robustness and reproducibility of the observed IL-1β expression pattern, as well as to corroborate the biological authenticity of this phenomenon. Consistent with previous findings, in our study, supplementation with SCFP led to varying degrees of reduction in the concentrations of the inflammatory factors IL-1β, TNF-α, IL-6, SAA, LBP, and HPT in the serum of cows. These findings indicate that supplementation with SCFP enhances the immune capacity of cows and reduces inflammatory responses.

Milk yield is the most direct indicator reflecting the production performance of dairy cows. The increased antioxidant capacity ultimately results in increased milk production in dairy cows. Numerous studies have shown that supplementation with SCFP can increase milk production in dairy cows (Erasmus et al. 2005; Robinson and Erasmus 2009; Poppy et al. 2012; Zaworski et al. 2014; Acharya et al. 2017). Feeding cows SCFP during the lactation period can increase milk production by 1.2–3.6 kg/d (Poppy et al. 2012; Dias et al. 2018; Carpinelli et al. 2021). Similar to the results reported in the literature, supplementation with SCFP in the present study positively influenced milk yield in the early to mid-lactation period (during the first 20 wk relative to parturition). Additionally, not only did milk production significantly increase during the period of SCFP supplementation (first 60 d relative to parturition), but milk production remained higher than that of the control group after SCFP supplementation was discontinued. This phenomenon could be due to the supportive effects of SCFP on the balance of composition and optimized function of the ruminal microbiota (Huang et al. 2021; Wang et al. 2023), as well as its ability to promote the host’s antioxidant (Dai et al. 2024) and anti-inflammatory capacity (Trevisi et al. 2014; Trevisi and Minuti 2018). Further research under more controlled conditions is warranted to explore the mechanism of the potential carryover effect of SCFP.

## Conclusion

Our findings indicate that supplementation with SCFP from the day of dry-off through early lactation in dairy cows increases the relative abundance of beneficial bacteria, reduces oxidative stress and inflammation, aids in maintaining ruminal microbial fermentation function on the day of parturition, and increases the production of VFAs. These effects contribute to increased milk yield in dairy cows. Moreover, owing to the cumulative benefits accrued during the SCFP supplementation period, milk production in dairy cows continues to improve even after SCFP is discontinued.

## Supplementary Material

skag056_Supplementary_Data

## Abbreviations

AIA: acid insoluble ash
ALB: albumin
ASVs: amplicon sequence variants
BHBA: β-hydroxybutyrate
DHI: Dairy Herd Improvement
EE: ether extract
GLU: glucose
GSH-Px: glutathione peroxidase
HPT: haptoglobin
LBP: lipopolysaccharide binding protein
LDA: linear discriminant analysis
LEfSe: linear discriminant analysis effect size
MDA: malondialdehyde
NEFA: non-esterified fatty acids
NH3-N: ammonia N
PCoA: principal coordinate analysis
SAA: serum amyloid A
SCFP: *Saccharomyces cerevisiae* fermentation product
SOD: superoxide dismutase
T-AOC: total antioxidant capacity
TBIL: total bilirubin
TC: total cholesterol
TMR: total mixed ration
TNFα: tumor necrosis factor-alpha
TP: total protein
VFA: volatile fatty acid
VIF: variance inflation factor.
