# Supplementary material for: Performance, blood parameters, ruminal fermentation and microbial community of dairy cows supplemented with Saccharomyces cerevisiae fermentation product from dry-off to early lactation
Source: J Anim Sci. 2026 Mar 4;104:skag056. doi: 10.1093/jas/skag056 (PMC13008330; doi:10.1093/jas/skag056)
Supplement: skag056_Supplementary_Data [file skag056_supplementary_data.docx]

Supplementary Table S1. Method Validation for IL-1β Assay: Linearity and Recovery

| ****Assay Parameter**** | ****Sample**** | ****Average (%)**** | ****Range (%)**** |
| --- | --- | --- | --- |
| **Linearity** | Serum (1:1 dilution, n=4) | 98 | 95–105 |
|  | Serum (1:2 dilution, n=4) | 95 | 90–97 |
|  | Serum (1:4 dilution, n=4) | 91 | 86–93 |
|  | Serum (1:8 dilution, n=8) | 84 | 81–91 |
| **Recovery** | Serum (Spiked, n=5) | 97 | 92–105 |
|  | EDTA Plasma (Spiked, n=4) | 93 | 85–95 |
